# Challenges, progress, and new directions in stem cell therapies: a new section launched in *Clinical and Translational Medicine*

**DOI:** 10.1186/s40169-015-0072-3

**Published:** 2015-09-16

**Authors:** Mariusz Z. Ratajczak, Xiangdong Wang

**Affiliations:** Stem Cell Institute at James Graham Brown Cancer Center, University of Louisville, 500 S. Floyd Street, Rm. 107, Louisville, KY 40202 USA; Zhongshan Hospital, Fudan University and Shanghai Institute of Clinical Bioinformatics, Shanghai, China

Every century is characterized by scientific achievements that become its historical landmarks and symbols. The previous century will be remembered for its significant discoveries relating to theoretical physics, chemistry, and biology, including stem cells. This latter discovery will be especially memorable for having opened the door to the development of regenerative medicine. There is no doubt that stem cell-based therapies will be regarded by future generations as a landmark achievement of the twenty-first century. While this is a huge challenge for medical science, there is hope that new therapies will be developed that enable several so-far-incurable diseases to be cured, prolonging life span and improving the quality of life. *Clinical and Translational Medicine* will keep pace with these new challenges by opening a new section dedicated to stem cell therapies, which will offer original peer-reviewed articles, reviews, editorials, commentaries, and perspectives. We will also welcome papers focused on bioethics, clinical regulations, clinical guidelines, and methodologies, and brief reports on experimental and clinical applications of stem cells. We were very lucky to recruit outstanding editorial board members for this section.

There are many journals dedicated to regenerative medicine. Nevertheless, we would like to be unique in this field in some way. Since the paradigms of science shift, and, as Albert Einstein once stated, “blind belief in authority is the greatest enemy of truth”, we would like to launch a new section that will provide a forum for new and challenging ideas and report progress in regenerative medicine. There are still many questions that need to be answered with hypothesis testing by independent groups. We are aware that our current work will require new generations of scientists not only to verify many of the current paradigms but also to carry forward progress in regenerative medicine in the coming decades.

Regenerative medicine has two main goals. The first is to employ stem cells efficiently and safely in therapies for injured organs and tissues. It is believed that, in the future, the transplantation of entire organs will be largely replaced by the transplantation of a suspension of stem cells directed to the given organ, alone or in combination with organic or synthetic scaffolds, which will perform the task of rebuilding the injured tissues. The second important and parallel goal of regenerative medicine is to develop strategies that will improve quality of life and longevity by improving the regenerative potential and proper functioning of adult stem cells residing in various organs.

Some of the most important challenges and topics that will be covered by this new section of *Clinical and Translational Medicine* are listed below.

*Search for the most efficient stem cell for clinical application* The search for stem cells that can be safely and efficiently employed for regeneration of damaged solid organs continues. Ideal for this purpose would be pluripotent stem cells, which, according to their definition, have broad potential to differentiate into cells belonging to the three germ layers [1]. For almost 20 years, there have been unsuccessful attempts to harness embryonic stem cells isolated from embryos, which has been controversial from an ethical point of view. A more promising source is induced pluripotent stem cells, which are generated by genetic modification of adult somatic cells. Unfortunately, both embryonic stem cells and induced pluripotent stem cells bear a similar risk of tumor formation after injection into the host [1, 2]. On the other hand, stem cells isolated from postnatal tissues, such as bone marrow, mobilized peripheral blood, umbilical cord blood, umbilical cord, placenta, fat, or epidermis, are the only stem cells that have been employed safely in the clinic so far. Since these are already committed to certain lineages, the question remains whether adult tissues also harbor other stem cells with much broader differentiation potential across germ layers that are multi- or pluripotent. These investigations and the search for such cells have prompted to challenge the accepted hierarchy of the stem cell compartment in adult tissues [1, 2].

*Paracrine*- *and extracellular microvesicle*-*mediated effects of stem cell therapies* While mesenchymal stem cells, cardiac stem cells, and hematopoietic stem cells are employed in the clinic, there has been a lack of convincing documentation for donor–recipient chimerism in the solid organs treated, and most studies indicate that a mechanism other than transdifferentiation of cells plays a role, most likely involving the paracrine effects of growth factors, cytokines, chemokines, bioactive lipids, and extracellular microvesicles released from cells employed as cellular therapeutics [3, 4]. These beneficial paracrine mechanisms could be harnessed more efficiently to regenerate damaged organs. Stem cells could be modified to secrete more of the paracrine factors that inhibit apoptosis in damaged tissues and enhance vascularization. One of the immediate and safe applications for induced pluripotent stem cells would be their application as cell lines for producing therapeutic extracellular microvesicles [3, 4].

*Molecular mechanisms involved in stem cell pluripotency, quiescence, proliferation, and differentiation* The number of stem cells isolated from adult tissues is usually very limited. In particular, the most primitive stem cells (from a developmental point of view) are present at very low frequencies in adult tissues. To overcome this problem, different ex vivo expansion strategies have been proposed to amplify the numbers of these cells so that they can be employed more efficiently in the clinic. Unfortunately, optimized expansion protocols for the most primitive stem cells have not been described so far, and during expansion these most primitive cells decrease in number due to their differentiation into more mature progenitors [5]. An efficient expansion strategy requires identification of the set of genes that balances pluripotency, multipotency, and stem cell self-renewal with differentiation. Unravelling the mechanisms that govern the quiescence of the most primitive stem cells in adult tissues is of primary importance in developing expansion strategies for these rare cells that will allow their application in the clinic [6]. An important additional goal of regenerative medicine is to develop strategies that enhance quality of life and longevity by improving the regenerative potential and proper functioning of adult stem cells residing in various organs. This could be achieved by application of anti-aging drugs, caloric restriction, or regular physical activity [7]. Thus, anti-aging strategies for stem cells will be one of the important topics of this new section.

*Mechanisms that govern stem cell trafficking, including mobilization and homing to damaged tissues* It is known that stem cells are endowed with migratory potential and that they are released from their niches in a process known as mobilization. In the reverse process, stem cells settle into niches during so-called homing. Mechanisms that govern mobilization and homing have been best studied so far in hematopoietic stem cells [1, 8–10]. However, evidence has accumulated that other types of stem cells, such as mesenchymal stem cells, also undergo mobilization into peripheral blood. Therefore, in order to enrich peripheral blood in circulating stem cells, more efficient mobilization protocols have to be developed. On the other hand, we need to identify the mechanisms and all the regulatory factors (e.g., chemokines, growth factors, bioactive lipids, extracellular nucleotides) that govern trafficking of stem cells in the body. It is also crucial to develop optimal strategies for harvesting stem cells and to establish protocols for their delivery to damaged tissues. Another important issue is delivery of stem cells using special scaffolds that accelerate organ regeneration.

*Clinical ethics, regulations, trials, protocols, and practices for stem cell therapies* All new experimental treatment strategies in regenerative medicine must ultimately be tested in the clinic. This approach requires dealing with ethical and regulatory issues and has to be based on solid clinical development and approval by appropriate board protocols. It is obvious that clinical application of stem cells that follows good medical practice guidelines will require unified procedures and protocols so that trials can be compared between different centers [5, 11, 12]. This will require communication and cooperation between investigators around the globe. Our section will provide such a forum for the exchange of ideas and unification of procedures.

## Conclusions

*Clinical and Translational Medicine* is launching a new section on stem cell therapies that will concentrate on experimental and clinical results for stem cell applications in regenerative medicine, stem cell isolation, and expansion, with a focus on stem cells isolated or derived from adult tissues, umbilical cord blood, umbilical cord, placenta, fat tissue, and myocardium. It will also deal with molecular mechanisms involved in stem cell pluripotency, quiescence, proliferation, and differentiation and will cover mechanisms that regulate stem cell trafficking, including their mobilization into peripheral blood and homing to damaged tissues. Moreover, it will cover paracrine- and extracellular microvesicle-mediated effects of stem cell therapies, clinical ethics, regulations, trials, protocols, and practices of stem cell therapies in regenerative medicine. We encourage investigators to submit their best work so that, if accepted, it will be immediately spread throughout the scientific community through the open access format of our journal. Our section will be wide open to new discoveries and challenging ideas and we would like to welcome and publish your best research.
